# Antibiotic resistance patterns of urinary tract pathogens in Turkish children

**DOI:** 10.1186/s41256-018-0063-1

**Published:** 2018-03-16

**Authors:** Suzan Gunduz, Hatice Uludağ Altun

**Affiliations:** 1 Department of Pediatrics, Private Batman Hospital, Gultepe mah. Palmiyem 1 sitesi D blok no: 13, Batman, Turkey; 2 Microbiologist and clinical Microbiologist, Ankara, Turkey

**Keywords:** Urinary tract infection, Resistance patterns, Uropathogens, Empirical treatment, Urinary culture

## Abstract

**Background:**

Knowledge of local antimicrobial resistance patterns is essential for evidence- based empirical antibiotic prescribing. We aimed to investigate the distribution and changes in causative agents of urinary tract infections in children and the resistance rates, and to recommend the most appropriate antibiotics.

**Methods:**

In this retrospective study, we evaluated causative agents and antimicrobial resistance in urine isolates from the positive community from September 2014 to April 2016 in a single hospital in Ankara, Turkey.

**Results:**

A total of 850 positive urine cultures were identified, of which 588 (69.2%) were from girls and 262 (30.8%) were from boys. Their mean age was 36.5 ± 45.0 months. The most common causative agent was *Escherichia coli* (64.2% of cases) followed by *Klebsiella pneumoniae* (14.9%). The overall resistance to ampicillin (62.6%), cephalothin (44.2%), co-trimoxazole (29.8%) and cefuroxime (28.7%) was significant. No resistance to imipenem was detected in the isolates. The least resistance was for amikacin, ceftriaxone, ciprofloxacin and cefepime (0.1, 2.4, 7.5 and 8.3%, respectively). Imipenem was the most active agent against *E. coli* followed by amikacin (0.2%), ceftriaxone (2.7%) and nitrofurantoin (5.1%). High resistance rates to nitrofurantoin were detected in *K. pneumoniae, Proteus* and *Enterobacteriae*.

**Conclusions:**

*E. coli* was the most common causative agent of urinary tract infection in children. Ampicillin, trimethoprim-sulfometaxazole, cephalothin and cefuroxim had the highest resistance rates against urinary tract pathogens in our center. For oral empirical antibiotherapy, cefixime is the most appropriate choice so as to include *Klebsiella strains.*

## Background

Urinary tract infection (UTI) is one of the most common bacterial disease in children; it is acquired by an estimated 3–5% of girls and 1% of boys and represent a significant source of exposure to antibiotics in the pediatric population [1–3]. The initial treatment of acute UTI is based on patient symptomatology and urinalysis without microbiological confirmations [1–3]. Early diagnosis and prompt antimicrobial treatment are required to minimize mortality including renal abscess formation, septicaemia, renal scarring, and even renal failures [4]. Moreover, they constitute a serious economic cost for countries. The economic impact may, however, be substantial because of the large number of acutely unwell children who present to primary care, additional diagnostic tests for structural abnormalities of the urinary tract, rare but serious complications of UTI, and the wider impact of antibiotic prescribing on bacterial resistance. Loss of time in school for children and loss of parental workforce loss are indirect costs [5, 6].

The initial choice of antibacterial therapy is based on knowledge of the predominant pathogen in the patient’s age group, antibacterial sensitivity patterns in the practice area, the clinical status of the patient, and the opportunity for close follow-up [2, 4].

The current American Academy of Pediatrics guideline for management of UTIs in febrile infants and young children suggests giving oral or parenteral (then changed to oral) antibiotics for 7–14 days. Ceftriaxone, cefotaxime, ceftazidime, gentamicin, tobramycin, and piperacillin are drugs of choice for parenteral therapy. By contrast, amoxicillin-clavulanate, sulfonamide (trimethoprim-sulfamethoxazole or sulfisoxazole), or cephalosporin (cefixime, cefpodoxime, cefprozil, cefuroxime axetil, or cephalexin) are recommended as oral agents for treating UTI [7, 8].

Studies of pediatric uropathogens indicate that resistance to common antibiotics is on the rise [9] and treatment of UTIs is becoming more difficult with time. Moreover, there are considerable geographic variations in bacterial patterns and resistance properties depending on local antimicrobial prescription practices [2, 8, 10, 11]. However, because of the evolving and continuing antibiotic resistance phenomenon, regular monitoring of resistance patterns is necessary to improve guidelines for empirical antibiotic therapy.

UTIs may demonstrate different epidemiological and etiologic features due to gender, age and region. So regional studies from different time periods are of great importance for better understanding of the disease, effective treatment and prevention of complications. Our goal was to analyze the aetiology and resistance patterns to antibiotics of urinary isolates in order to evaluate options for empirical antibiotic therapy of UTI in childhood. This information may assist in optimising empirical antibiotic management of this common infectious disease.

## Methods

### Patients and bacterial isolates

The present study is retrospective, non-randomised and convenient sampling. All urine cultures from September 2014 to April 2016 were reviewed from children younger than 16 years of age admitted to the Department of Pediatrics of Turgut Ozal University with suspected UTI. Because of reviewing medical files of patients, we did not take informed consent.

Urine samples were collected following standard perineoscrotal hygiene by a nurse into sterile urine bags in incontinent patients, and from continent patients midflow urine samples were obtained.

### Antimicrobial susceptibility

Urine samples were sent to the laboratory where they were inoculated using a 4 mm caliberloop on culture media containing eosin methylene blue (EMB) agar and 5% sheep blood agar plate, and incubated at 37 **°**C for 18–24 h. Conventional methods (colony morphology, Gram stain, MVC reactions) and Phoenix TM 100 (Becton Dickinson, Franklin Lake, NJ, USA) fully automatic identification system was used for the definition of isolates. In compliance with CLSI (Clinical and Laboratory Standards Institute) criteria, a disc diffusion method was used to perform in vitro antimicrobial susceptibility tests against trimethoprim-sulfometaxazole (TMP-SMX), cefotaxime, ceftriaxone, cefuroxime axetil, ciprofloxacine, amikacin, amoxicillin-clavulanate, gentamicin, amoxicillin and nitrofurantoin [12]. Although ciprofloxacin is not commonly used in paediatric patients, it was included in the analysis. The use of ciprofloxacin in childhood UTIs is limited to infections caused by *Pseudomonas aeroginosa* and other multidrug-resistant. All antibiotics included in this study cover the appropriate organisms and are used in the treatment of UTIs with varying degrees of success. Only the first positive urine culture obtained per patient on admission was included in the analysis to eliminate any possibility of recurrence. Polymicrobial cultures and cultures with multidrug-resistant uropathogens were not included in the analysis for not including nosocomial infections and contaminations. Cultures with *Candida* growth were excluded. Significant growth was evaluated as ≥10^5^ colony-forming units (CFU)/ml.

To analyze resistance to antibiotics for different ages, subjects were divided into three age groups: Group I, ≤12 months; Group II, 13–60 months; and Group III, > 60 months.

### Statistical analysis

For the evaluation of the study data SPSS (Statistical Package for the Social Sciences, version 23.0 for Windows, SPSS® Inc., Chicago, IL, USA) statistical analysis program was used. Frequency, and mean ± standard deviation (SD) of the data were provided. Median resistance to antimicrobial agents was calculated for each causative pathogen. *P* < 0.05 was considered statistically significant.

## Results

A total of 1290 urine samples were reviewed and 850 urine cultures were eligible. Mean patient age was 36.5 ± 45.0 months (range 1 to 208 months). Five hundred and eighty-eight pathogens (69.2%) were isolated from girls and 262 (30.8%) were from boys.

According to the age of the patients, the numbers of collected isolates were as follows: 415 (48.8%) were obtained from patients in Group I, 226 (26.6%) from patients in Group II and 209 (24.6%) from patients in Group III.

The most common causative agent both in total and among different age groups was Escherichia coli (64.2% of cases) followed by Klebsiella pneumoniae (14.9%), Enterococcus (5.4%), Klebsiella oxytoca and Proteus mirabilis (3.9%) and Enterobacter spp. (1.8%). Resistance to ampicillin (62.6%), co-trimoxazole (29.8%) and cefuroxime (28.7%) in all isolates was significant.

Amikacin was the most susceptible agent against E. coli (0.2% resistant isolates), followed by ceftriaxone (2.7%), nitrofurantoin (5.1%) and ciprofloxacin (7.9%). Amikacin, imipenem and ceftriaxone were also the most susceptibleagents against K. Pneumoniae, however none of the isolates were found to be resistant to amikacin and imipenem, and only a small number resistant to ceftriaxone. Table 1 shows the frequency of resistance to selected antibiotics.

**Table 1:** Resistance rates (%) of the most frequent urinary tract pathogens against tested antibiotics

Antibiotic	*E. coli*	*K. pneumoniae*	*Enterococcus*	*K. oxytoco*	*Proteus* *spp.*	*Enterobacteriae*	Overall resistance
Ampicillin	58.2	98.4	80.0	97.0	45.5	80.0	62.6
Co-trimoxazole	31.5	30.7	NT	15.2	30.3	13.3	29.8
Gentamicin	10.1	16.5	NT	12.1	3.0	6.7	10.8
Ciprofloxacin	7.9	7.1	NT	7.1	6.1	6.7	7.5
Ceftriaxone	2.7	1.1	NT	7.7	–	–	2.4
Nitrofurantoin	5.1	49.1	10.9	17.9	97.0	57.1	17.1
Amikacin	0.2	–	–	–	–	–	0.1
Amoxicillin-clavulanate	16	21.3	NT	18.2	9.1	60	17.6
Cefuroxime	27.6	36.8	NT	36.4	–	46.7	28.7
Ceftazidime	10.7	21.4	NT	–	–	NT	10.7
Cefepime	8.1	12.6	NT	–	1.0	NT	8.3
Imipenem	–	–	NT	–	–	–	–
Cephalotin	45.2	41.1	NT	48.4	14.3	80.0	44.2

*NR*= No resistance

*NT*= Not tested

As the most prevalent uropathogen, the resistance of E. coli is presented in more detail. Table 2 shows the age-based distribution of resistance of E. coli to various antibiotics. The highest overall resistance was seen for ampicillin, cephalotin, TMP-SMX and cefuroxime, with other antibiotics showing some resistance.

**Table 2:** Percentage of *E. coli* and various drug resistance rates against *E. coli* according to age groups

	1–12 months	13–60 months	> 60 months
Amikacin	–	–	0.6
Gentamicin	11.3	12.8	5.7
Ciprofloxacin	8.3	7.1	8.2
Cefuroxime	**27.3**	**32.5**	**23.1**
Ceftazidime	4.5	15.8	13.3
Ceftriaxone	2.1	5.0	1.5
Cephalotin	**46.2**	**47.3**	**41.8**
Ampicillin	**56.3**	**57.7**	**61.6**
TMP-SMX^a^	**30.7**	**31.4**	**32.7**
Nitrofuratoin	6.7	3.9	3.9
Amoxicillinclavulanate	16.1	17.9	13.9

The resistance rates exceeding 20% were in boldface

-= No resistance

*= trimethoprim-sulfometaxazole

## Discussion

Our data confirm that E. coli is still the most common single organism causing UTIs in children under 18 years of age; however, other bacterial strains are now more frequently isolated from UCs (urine culture) than in the past [1, 13, 14]. However, the second and the third most common microorganisms differ in various studies and similar results were observed in studies performedin our country [6, 15–20]. In a study from the same province, Catal and his colleaques demonstrated an increasing rate of Klebsiella spp. detected in urine culture in the years 2000 and 2006, of 7.2% and 18%, respectively [15]. The percentage of Klebsiella spp. detected in urine culture in the present study is in accordance with other Turkish studies [15–20] and is worrisome.

In children with serious clinical manifestation, hospitalization and empirical parenteral antibiotherapy is required [1–5]. As reported in a previous study [21], non-E. coli pathogens are more resistant to most antimicrobial agents (amoxicillin-clavulanate, cephalosporin, nitrofurantoin and amikacin); thus, empirical antibiotic therapy may not always be appropriate. We have to take into consideration the ineffectiveness of oral empirical antibiotherapy for UTI because of increasing Klebsiella strains. Increasing cases of Klebsiella spp. may increase hospitalization and parenteral antibiotherapy and also cost. For an antibiotic to be considered a first line empirical treatment for urinary tract infection, resistance should not exceed 20% in the most likely infecting strain [22]. Bryce and colleagues showed that this threshold has been reached for many first line antibiotics used for pediatric E. coli urinary tract infection. Within countries in the OECD (Organisation for Economic Co-operation and Development), half of all isolates were resistant to ampicillin, a third to co-trimoxazole, and a quarter to trimethoprim. Resistance was substantially greater in non-OECD countries. Data confirmed the group’s previous review suggesting that previous antibiotic use in primary care increased the subsequent risk of E. coli resistance to that particular antibiotic [10]. In accordance with the present study, worldwide rates of resistance of E. coli to ampicillin are the highest and nitrofurantoin rates the lowest [10]. In a meta-analysis from Turkey, Aykan and her colleagues analyzed antibiotic resistance changes of E. coli between 2002 and 2012 [23]. Though the resistance rate changed through the years, we documented lower resistance rates of E. coli to ampicillin, co-trimoxazole, ceftriaxone, amikacin, amoxicillin-clavulanate and the same resistance rates for gentamicin, nitrofurantoin and ciprofloxacin in comparison to Aykan’s meta-analysis [23].

Appropriate treatment requires information regarding the susceptibility patterns of the current bacteria in order to give effective antibiotics in a timely manner [24, 25]. Health care providers must be aware of the resistance patterns of uropathogens in their practice area and prescribe empirical antibiotics.

Higher resistance rates were detected against frequently used medications such as ampicillin, TMP-SMX and cefuroxime, which are preferred because of their oral intake. These higher resistance rates suggest that these antibiotics should not be selected for empirical treatment in our province. The reason for these higher resistance rates might be attributed to a long-termpreference for these antibiotics in general medical practice, and alteration in resistance rates with time.

Oral UTI treatment is as efficient as parenteral treatment [25]. Hospitalization or parenteral treatment is indicated in infants younger than 2 months with toxic appearance who can not take fluid or medication by the oral route, in immune deficiencies and when there is social justification [1, 2, 5].

Cephalosporins are increasingly prescribed to treat this infection. Ceftriaxone was used as marker of the 3rd- generation cephalosporins. Oral 3rd-generation cephalosporins such as cefixime are as effective as parenteral ceftriaxone against a variety of gram negative organisms other than Pseudomonas spp., and these medications are considered to be the treatment of choice for oral outpatient therapy [1, 4, 26]. Because of convenient dosing (one to two doses administered daily) and antibacterial activity, this oral antibiotic has been used at many centers and for many years for outpatient treatment of uncomplicated community-acquired UTIs in children [1, 4, 26].

Though nitrofurantoin is an effective oral antibiotic, it should not be used routinely in children with febril UTI, because it does not achieve significant renal tissue levels [1]. The results of the present study showed cefixime as an oral 3rd generation cephalosporin to be the most effective oral antibiotic for UTI. In medical practice, pediatricians prescribe mostly cefixime for UTI in children in recent years in our province. This is an appropriate choice for empirical treatment. We have to take into consideration the increasing resistance of uropathogens to this antibioti in the near future. Antimicrobial resistance is an internationally recognised threat to health. Not only uropathogens but also all pathogens will develop resistance to antimicrobials. Rational antibiotic use is essential in medical practice. This information may assist in optimising empirical antibiotic management of this common infectious disease.

Our study has the following limitations. Being retrospective, it suffers from the well-known limitations of that experimental design. Subjective bias, however, is mitigated by its reliance on laboratory data alone. A second limitation relates to in vitro susceptibility testing, which may not translate to specific clinical outcomes. We did not perform follow-up on the patients, and do not have information about the outcomes of the patients or any further diagnostic testing that was performed.

## Conclusion

E. coli was the most common causative agent of UTI in children in our center. A high rate of resistance was found to first and second generation cephalosporins, ampicillin, amoxicillin clavulanate and TMP-SMX, which are the first line agents in childhood UTI. The increasing percentage of Klebsiella spp. recognised in urinary cultures is worrisome. For oral empirical antibiotherapy, cefixime is the most appropriate choice so as to include Klebsiella strains. This study will be useful for physicians in Turkey to improve appropriate empirical treatment for UTI. Though there are universal guidelines, we suggest that empirical antibiotic selection should be done based on the local prevalence of bacterial organisms and antibiotic sensitivities rather than on universal guidelines.

## Abbreviations

CFU: Colony-forming units
CLSI: Clinical and Laboratory Standards Institute
EMB: Eosin methylene blue
OECD: Organisation for Economic Co-operation and Development
TMP-SMX: Trimethoprim-sulfometaxazole
UC: Urine cultur
UTI: Urinary tract infection
